# Leishmaniasis Transmission Risk at the Forest‐Peridomestic Interface in an Area of Southern Sinaloa, Mexico: Entomological, Molecular, and Climatic Evidence

**DOI:** 10.1155/japr/5071505

**Published:** 2026-06-16

**Authors:** Juan José Ríos Tostado, Hipólito Castillo Ureta, José Israel Torres Avendaño, José Marcial Zazueta Moreno, César Enrique Romero Higareda, Luz Isela Peinado Guevara, Annete Itzel Apodaca Medina, Edith Hilario Torres Montoya

**Affiliations:** ^1^ Postgraduate Programme in Biological Sciences, Faculty of Natural and Exact Sciences, Autonomous University of Sinaloa, Culiacán Rosales, Sinaloa, Mexico, uas.edu.mx; ^2^ Health Science Department, Autonomous University of Occidente, Culiacán Rosales, Sinaloa, Mexico

**Keywords:** *Bichromomyia olmeca olmeca*, climate change, *Culicoides furens*, entomological surveillance, *Leishmania* spp., neglected tropical diseases, trophic spectrum

## Abstract

Leishmaniasis, caused by *Leishmania* spp. and transmitted by phlebotomine sand flies, affects almost 1,000,000 people annually across more than 90 countries. In Mexico, growing evidence of locally acquired transmission in northwestern states makes ecoepidemiological work increasingly urgent. We aimed to conduct a 2‐year ecoepidemiological study in four rural communities of Sinaloa, Mexico, to identify vectors, determine their food sources, detect *Leishmania* DNA, and relate it to climatic factors. CDC light traps were operated bimonthly along a peridomestic‐to‐forest gradient; dipterans were identified morphologically, *Leishmania* spp. DNA confirmed by PCR‐RFLP, and habitat differences tested with Kruskal–Wallis and Spearman rank correlation (*α* = 0.05). We collected 9984 dipterans over 12 sampling rounds; sylvatic sites accounted for 81.5% of captures, peaking in September and November. *Culicoides furens* (Ceratopogonidae) was numerically dominant. Three sand fly species were recovered: *B*. *olmeca olmeca*, *M*. *cayennensis maciasi*, and *P*. *texana*. *Leishmania* spp. DNA was detected in 7 of 35 *C*. *furens* pools; no sand fly tested positive. *C*. *furens* fed primarily on *Homo sapiens*; *M*. *cayennensis maciasi* fed on *Canis lupus familiaris* in peridomestic settings. Rainfall was the strongest predictor of abundance (Spearman rho up to 0.824, *p* < 0.01); wind speed showed an inverse relationship. Repeated detection of *Leishmania* DNA in *C*. *furens* suggest the hypothesis that this species contacts infected reservoirs, though experimental vector competence studies remain needed. The first record of *B*. *olmeca olmeca* for Sinaloa carries clear epidemiological implications. The forest–community interface is the dominant exposure zone, and risk is likely to intensify with rising temperatures. Priority actions include: experimentally evaluating the vector competence of *C. furens*, and intensifying sand fly surveillance to clarify the role of local dipteran fauna in *Leishmania* transmission dynamics.

## 1. Introduction

*Leishmania* (Kinetoplastida: Trypanosomatidae) is transmitted to vertebrates by the bite of female phlebotomine sand flies (Diptera: Psychodidae) called *Lutzomyia* in the Americas and Caribbean and *Phlebotomus* in Europe, Asia, and Africa. The disease is a complex zoonosis whose dynamics hinge on the interaction of parasite, competent vector, vertebrate reservoir, and human host in specific local ecosystems [1]. WHO lists leishmaniasis among the neglected tropical diseases of greatest global impact, with 700,000–1,000,000 new cases each year in more than 90 tropical and subtropical countries [2]. The burden falls disproportionately on rural communities in poverty, particularly those with occupational exposure to forest environments or those caught in the path of agricultural expansion into endemic zones [3].

Three clinical forms occur in humans. Cutaneous leishmaniasis (CL) is most common, presenting as one or more dermal ulcers at the bite site. Mucocutaneous leishmaniasis (MCL) is more severe, with progressive destruction of the nasopharyngeal mucosa. Visceral leishmaniasis (VL), or kala‐azar, is the most dangerous form and is fatal if left untreated [4, 5]. The genus *Leishmania* comprises more than 20 species pathogenic to humans, divided into two subgenera: *Leishmania* (*Viannia*), which develops in the hindgut of the vector (suprapylarian), and *Leishmania* (*Leishmania*), which develops in the midgut (peripylary). In Latin America, the clinically most relevant species are *Leishmania mexicana*, *Leishmania braziliensis*, *Leishmania panamensis*, *Leishmania amazonensis*, and *Leishmania infantum* (synonym *Leishmania chagasi*) [6, 7].

Sand flies (Psychodidae: Phlebotominae) are the only insects proven to transmit *Leishmania* spp. under natural conditions. They are small dipterans, no larger than 3.5 mm, with hairy, pointed wings. They are most active at dusk and at night, flying short distances and making no noise while flying. Females must take blood meals to complete ovarian development, whereas males subsist on plant sugars [8, 9]. In Latin America and the Caribbean, the genus *Lutzomyia* holds more than 400 described species, though only a small fraction have been confirmed or implicated as vectors. For Mexico and Central America, the most important include *Bichromomyia olmeca olmeca* (formerly *Lutzomyia olmeca olmeca* [Vargas and Díaz‐Nájera, 1959]), the primary vector of *L. mexicana* in Yucatán, *Lutzomyia cruciata* and *Lutzomyia diabolica* in arid and semiarid northern zones, and *Lutzomyia longipalpis*—the vector of *L. infantum,* responsible for visceral leishmaniasis hotspots in the southeast [10–12]. Sand fly ecology is tightly tied to high relative humidity and substrates with abundant organic matter (leaf litter, humus‐rich soils, rock crevices, tree bases) for larval development, plus reliable access to blood. Temperature, rainfall, and wind speed are the main abiotic factors controlling seasonal population dynamics, with peaks during the warmest, wettest months [13–15].

Leishmaniasis is fundamentally a zoonosis maintained by wild mammals. In Latin America, rodents of the genera *Oryzomys*, *Proechimys*, and *Heteromys*, marsupials of the genus *Didelphis*, and other wild mammals have been identified as hosts for *Leishmania* parasites. In the peridomestic cycle, the domestic dog (*Canis lupus familiaris*) is the main reservoir of *L. infantum* and bridges the gap between the sylvatic cycle and human transmission [6, 16, 17].

Characterizing trophic spectrum (identifying the vertebrates from which blood‐feeding insects have fed) is an important tool in evaluating transmission risk. PCR‐RFLP analysis of the mitochondrial *CYTB* gene has allowed direct identification of host preferences in wild sand fly populations, showing that a single species can feed on both wild and domestic animals depending on local host availability [18–20]. This opportunism increases the likelihood of epidemiological bridging between sylvatic and domestic cycles, particularly in areas with fragmented habitat [21].

In Mexico, active cutaneous and visceral leishmaniasis transmission has been documented across a wide geographic range, though more than 80% of registered cutaneous cases are concentrated in southeastern states: Campeche, Tabasco, Quintana Roo, Chiapas, Yucatán, and Veracruz. Visceral cases are similarly concentrated in the south (Chiapas, Guerrero, Oaxaca, Morelos, Puebla), with only sporadic reports from northwestern states such as Sinaloa [22–24]. Official records from the Dirección General de Epidemiología show 600–1200 CL cases per year between 2015 and 2022, a figure that likely understates the true burden by five to tenfold, given limited access to confirmatory diagnosis in rural endemic areas [24]. Entomological and clinical data are especially scarce in the northwest, where formal surveillance has lagged well behind the southeast.

Certain ecological conditions predispose particular landscapes to transmission: lowland deciduous forest, mangrove swamp, and riparian vegetation; moderate to high temperatures and humidity; surface water availability; and the co‐occurrence of wildlife that can serve as blood meal sources [22, 25]. Deforestation and the expansion of agriculture disrupt landscape structure and bring wild sand fly populations into contact with humans and domestic animals that lack acquired immunity [26, 27]. The extensive mangrove systems of northwestern Mexico (a known ceratopogonid and phlebotomine hábitat) occur alongside active fishing and mango farming communities, a configuration that represents a genuine but poorly explored risk scenario.

Sinaloa was long considered outside the endemic range for leishmaniasis, but this view has been revised. *Leishmania* spp. DNA has been detected in sand flies from peridomestic settings in rural Sinaloan communities, and autochthonous CL has been diagnosed in both humans and dogs with no travel history to high‐endemicity areas [28–30]. No targeted vector control program for sand flies exists in the Mexican Pacific Northwest, making the generation of local transmission data an immediate practical need.

Climate change compounds this concern. Niche models based on temperature, precipitation, and vegetation cover project a 15%–40% expansion of suitable sand fly habitat in Mexico and Central America between 2040 and 2080, depending on the emissions scenario [31–33]. A 2°C rise in mean temperature (consistent with IPCC RCP4.5 and RCP8.5 projections) would shorten the extrinsic incubation period of *Leishmania* in its vector, potentially raising transmission efficiency in areas currently of low endemicity such as northwestern Mexico [34–38]. In this study, conducted over 2 years in four rural communities of southern Sinaloa, Mexico, we addressed the following specific objectives: (i) to identify and characterize the dipteran fauna (Ceratopogonidae and Psychodidae) along the peridomestic‐to‐forest gradient; (ii) to detect *Leishmania* spp. DNA in collected specimens; (iii) to determine the trophic spectrum of epidemiologically relevant species; (iv) to assess associations between climatic variables and dipteran abundance across habitats and seasons.

## 2. Materials and Methods

### 2.1. Study Area

**Figure 1:**
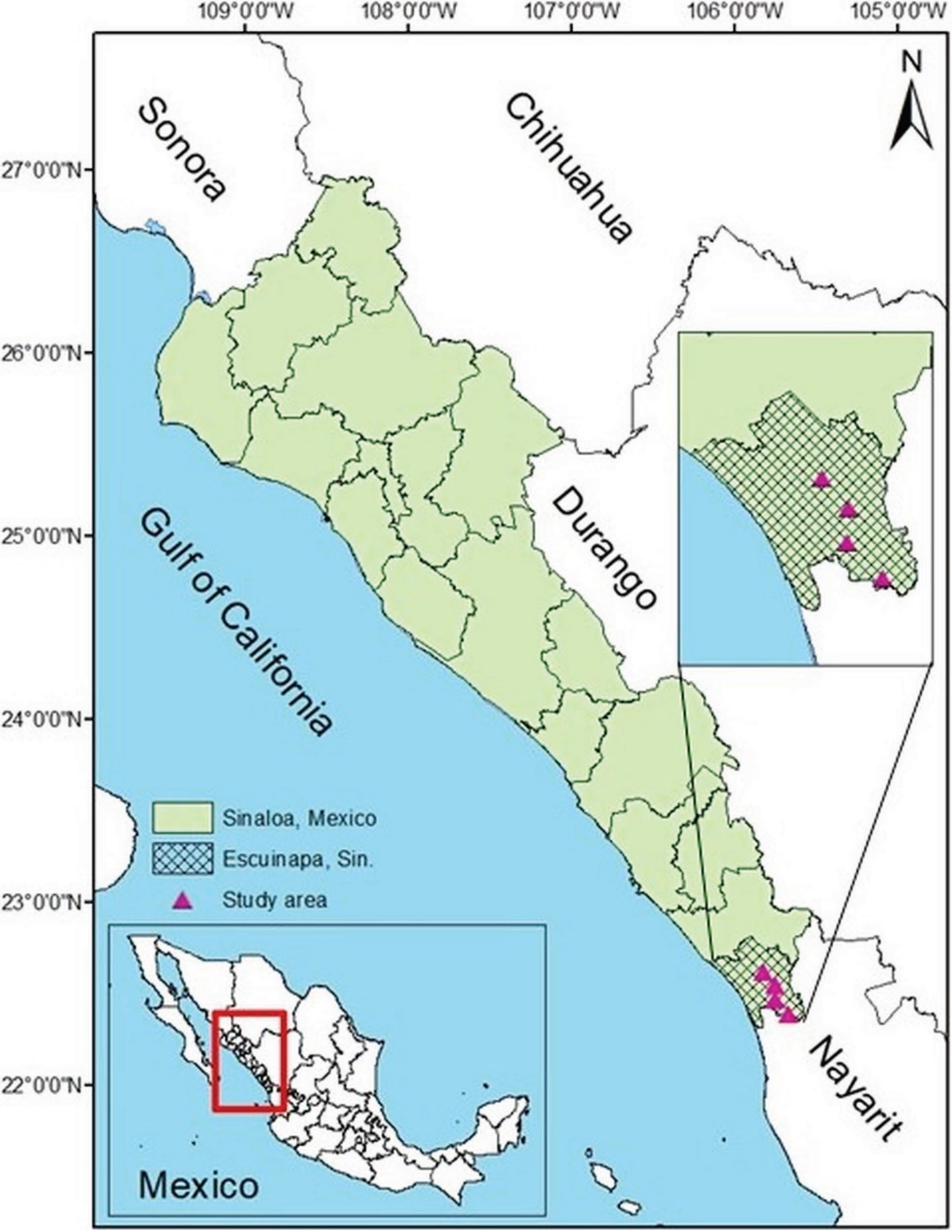
Geographical location of the sampling sites in southern Sinaloa, Mexico.

The study was conducted in four rural fishing and agricultural communities (Tecualilla, Ejido La Campana #1, Ojo de Agua de Palmillas, and Copales) within the municipality of Escuinapa de Hidalgo in southern Sinaloa, México (22°44 ^′^19.68″ N, 105°43 ^′^47.53″ W). The municipality borders Nayarit to the South, El Rosario to the North, the Pacific Ocean to the West, and the Sierra Madre Occidental to the East (Figure 1) [39]. Elevation is 22 m a.s.l. The climate is warm subhumid with an annual mean temperature of 26°C (maxima of 40°C–42°C) and strongly seasonal rainfall: < 20 mm/month from January to May and up to 875 mm accumulated in June through December (Climate Data Online). Dominant vegetation types are xerophytic scrubland, buffelgrass pasture (*Cenchrus ciliaris*), and well‐preserved mangrove areas; these habitats are recognized breeding grounds for nematoceran dipterans of medical importance [40, 41].

### 2.2. Eligibility Criteria for Study Site Selection

The selection of communities and sampling sites was guided by epidemiological, ecological, operational, and social criteria, consistent with the six‐category decision framework proposed by Guillot et al., [42] for sentinel site selection in vector‐borne disease surveillance. The municipality of Escuinapa de Hidalgo was identified as a priority surveillance zone based on prior evidence of local *Leishmania* transmission, including documented autochthonous canine leishmaniasis and detection of *L. mexicana* DNA in dipterans from the area [29, 30]. Its geographic position (a transition zone between the Pacific coastal wetlands of Marismas Nacionales and the foothills of the Sierra Madre Occidental) provides the landscape mosaic favorable for both ceratopogonid and phlebotomine development. The four selected rural communities are primarily engaged in fishing, seasonal agriculture, and mango cultivation, resulting in a fluctuating workforce from southern states where leishmaniasis prevalence is considerably high, representing an additional transmission risk. Their location near the Nayarit state border further justified their inclusion to ensure spatial representativeness of the sentinel network. Community willingness to participate was essential for sustaining the 2‐year longitudinal study. Within each community, four sampling sites were established along the peridomestic‐to‐forest gradient described by da Silva‐Chagas et al., [43]: two peridomestic sites and two forest sites at 100 and 300 m from the village perimeter. The 100 and 300 m distances bracket the active flight ranges of both target genera: *Culicoides* spp. typically disperse 200–500 m from breeding sites under natural conditions, whereas phlebotomine sand flies rarely exceed 200–300 m from larval habitat [8]. This gradient therefore captures both the zone of maximal human‐vector contact at the village edge and the deeper forest zone where sand fly abundance is highest. Sites were selected using the *worst-case transmission scenario* criterion, targeting microhabitats with decomposing organic matter, standing water suitable for dipteran oviposition, and structurally diverse vegetation offering the microclimatic conditions required for vector establishment and reproduction.

### 2.3. Collection of Nematoceran Dipterans

Sampling was performed bimonthly (1 round every 2 months) for a total of 12 rounds per locality and 48 sampling events across the four communities (July 2022–June 2024) with 192 trap‐events and 384 total trap‐hours. CDC‐type light traps were placed at a height of 1.5 m at preselected sites. Supporting Information 1 shows the locations where the traps were placed in the four communities. Traps were operated during the full‐moon phase, with 2‐hour twilight capture sessions whose exact timing followed the monthly solar calendar. Temperature (°C), relative humidity (%), and wind speed and direction were measured at each site with calibrated portable instruments, the precipitation averages were taken from the electronic portal of the Comisión Nacional del Agua (https://smn.conagua.gob.mx/). Specimens that could not be identified due to morphological damage were excluded.

### 2.4. Morphological Identification

Specimens were identified using diagnostic features including male and female genital morphology, oral apparatus structure, palp arrangement, wing venation, and wing pigmentation [44–48]. Absolute and relative frequencies were calculated for each taxon; spatiotemporal distributions were tabulated by locality, trap site, and sampling period.

### 2.5. Molecular Detection of *Leishmania* spp.

Genomic DNA was extracted from macerated tissue using a Wizard SV Genomic DNA Purification System (Promega Corporation, Madison, Wisconsin, United States) per the manufacturer′s instructions, resuspended in nuclease‐free water, and stored at −20°C. *Leishmania* spp. detection used PCR to amplify a 300–350 bp fragment of the ITS1 region of rRNA with primers LITSR (5 ^′^‐CTGGATCATTTCCGATG‐3 ^′^) and L5.8S (5 ^′^‐TGATACCACTTATCGCACTT‐3 ^′^) [49] in GoTaq Green Master Mix (Promega). Cycling conditions: 35 cycles of 94°C/40 s, 53°C/30 s, 72°C/60 s, with a final extension of 72°C/7 min. Products were visualized on 1% agarose stained with GelRed (GelDoc system, Bio‐Rad). Positive products were digested with *HaeIII* and separated on 2% agarose using a 100 bp ladder (Promega). The RFLP only served to confirm that the amplicon was *Leishmania* spp., and that identification at the species level would require sequencing which was not possible to perform. Strain MHOM/MX/UAY68 (*L. mexicana*) served as a positive control, and each PCR run included a negative control (nuclease‐free water substituted for template DNA) to monitor for cross‐contamination. To prevent cross‐contamination, DNA extraction, PCR setup, and amplicon analysis were performed in physically separated areas. Disposable gloves and filtered pipette tips were used throughout. Surfaces were decontaminated with 10% bleach and UV‐irradiated before each sesion. Only female *Culicoides furens* were processed for molecular analysis, as females are the sole blood‐feeding sex and the epidemiologically relevant sex for *Leishmania* detection; males were excluded from all pools. Because capture yields varied considerably across sampling events, pool sizes were not uniform, ranging from 1 to approximately 100 females per pool depending on locality, habitat, and sampling round. Minimum infection rates (MIRs) were calculated as: MIR  =  (number of positive pools/total number of females analyzed) × 1000 (CDC, 2025). The MIR formula was selected for its suitability with pools of unequal size, providing a conservative minimum estimate of infection prevalence.

### 2.6. Determination of Trophic Spectrum

A 358 bp fragment of the mitochondrial *CYTB* gene was amplified using primers BM1 (5 ^′^‐CCC TCA GAA TGA TAT TTG TCC TCA‐3 ^′^) and BM2 (5 ^′^‐CCA TCC AAC ATC TCA GCA TGA TGA AA‐3 ^′^) in GoTaq Green Master Mix on a T100 thermocycler (Bio‐Rad), with 35 cycles of 94°C/1 min, 65°C/30 s, 72°C/30 s, and a final extension of 72°C/7 min. PCR products were digested with *Aci*I, *Alu*I, *Hae*III, and *Rsa*I per manufacturer protocols and visualized on 1% agarose with GelRed [18].

### 2.7. Statistical Analysis

Data were organized in Microsoft Excel 2021. Differences in dipteran abundance among localities, habitats, and trap sites were tested with a three‐factor Kruskal–Wallis test (IBM SPSS Statistics v.22.0), as data did not meet normality assumptions (Shapiro–Wilk, *p* < 0.05). Environmental correlates of vector abundance were assessed with Spearman rank correlation (*r*_s_). Although a generalized linear mixed model (GLM/GLMM) would represent the statistically optimal approach for count data with repeated‐measures structure, nonparametric tests were retained given the limited sample size, the high proportion of zero counts in phlebotomine captures, and the exploratory‐descriptive objective of the study. Trophic spectrum differences by season, site, and host category were evaluated with Fisher′s exact test, which is more appropriate than chi‐square for small Psychodidae sample sizes. *Leishmania* positivity was expressed as percentage with 95% CI. Statistical significance was set at *α* = 0.05 [50]. The complete statistical workflow, including software names and versions, is presented in Supporting Information 2.

### 2.8. Ethical Aspects of the Study

(1) The entomological sampling was carried out under the premise that no mosquito species is listed as endangered or subject to protection under the Official Mexican Standard NOM‐059‐SEMARNAT‐2010. On the contrary, due to their great adaptability, abundance, and role as disease transmitters, they are considered pests or vectors of public health. Therefore, in Mexico, no permit is required for this type of collection. (2) This study did not require the direct manipulation or sampling of any mammals, humans, birds, reptiles, etc. The trophic spectrum was determined directly from DNA extracted from diptera. The human DNA identified in trophic spectrum analyzes was handled solely for aggregated epidemiological purposes, with no individual person identification. (3) Verbal informed consent from community members in each locality for peridomestic sampling were documented through community assembly records

### 2.9. Statement of AI Use

Grammar and English‐language editing, as well as editing of the figures and tables were assisted by Claude Sonnet 4.6. (Anthropic, San Francisco, California, United States; claude.ai), an artificial intelligence language model. All content, interpretations, and conclusions remain solely the responsibility of the authors.

## 3. Results

### 3.1. Frequency and Spatiotemporal Distribution of Dipterans

A total of 9984 dipterans of epidemiological relevance were collected across 12 bimonthly sampling rounds (July 2022–June 2024), total number díptera collected is shown in Supporting Information 3. Sylvatic environments predominated, accounting for 81.50% of all captures, whereas peridomestic sites contributed the remaining. At the community level, Copales yielded the highest number of specimens (*n* = 3715), whereas Ejido La Campana recorded the fewest (*n* = 1214). Within peridomestic environments, Tecualilla (*n* = 785) and Copales (*n* = 643) presented the greatest capture rates, indicating a comparatively elevated human–vector contact interface in these two communities. Regarding taxonomic composition, other diptera constituted the numerically dominant component (*n* = 9157; 91.7% of total captures). Among medically relevant families, Ceratopogonidae (*n* = 648; 6.5%) far exceeded Psychodidae (*n* = 23; 0.2%), with both target families together representing less than 7% of the total collection. Tecualilla accounted for the largest proportion of Ceratopogonidae (62.7%), whereas Copales concentrated the majority of Psychodidae specimens (65.3%). These distributional patterns are summarized in Table 1.

**Table 1:** Number of Diptera collected by locality and hábitat type, and distribution of Ceratópogonidae and Psychodidae across sampling sites.

Locality	Habitat (% of total per habitat type)			Diptera families (% of family total)		
	Peridomestic *n* (%)	Sylvatic *n* (%)	Habitat Total	Ceratopogonidae *n* (%)	Psychodidae *n* (%)	Other Diptera
Tecualilla	785 (42.4)	2650 (32.6)	3435	406 (62.7)	7 (30.4)	3,022
Ej. La Campana	300 (16.2)	914 (11.2)	1214	16 (2.5)	1 (4.3)	1,197
Palmillas	122 (6.6)	1498 (18.4)	1620	13 (2.0)	0 (0)	1,607
Copales	643 (34.8)	3072 (37.8)	3715	213 (32.8)	15 (65.3)	3,487
Total	1850 (100)	8134 (100)	9984	648 (6.5)	23 (0.2)	9313 (93.3)

*Note:* Percentages in the hábitat columns represent each locality′s contribution to the total per habitat type (peridomestic total = 1850; sylvatic total = 8134). Percentages in the Diptera family columns represent each locality′s contribution to the total of that family across all sites (Ceratopogonidae total = 648; Psychodidae total = 23).

**Figure 2:**
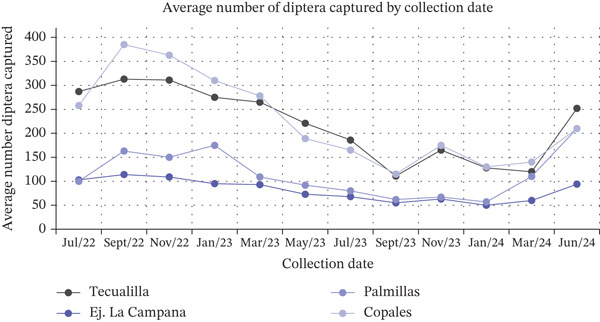
Mean dipteran captures per locality over 12 bimonthly sampling periods (July 2022–June 2024). Vertical dashed line marks the start of the second sampling year.

Abundance fluctuated seasonally throughout the monitoring period (Figure 2), with consistent peaks in September and November of both 2022 and 2023. Copales sustained the highest mean per sampling event (mean = 309.58 specimens), whereas Palmillas was lowest (mean = 135.00).

Within Ceratopogonidae, three species were identified: *C. furens* (Poey, 1964), *Culicoides insignis* (Lutz, 1913), and *Culicoides segnis* (Campbell and Pelham‐Clinton, 1960) (Supporting Information 4). The key distinguishing characters used in identification were: *C*. *furens* shows a distal white macula in cell R3 without a notch plus prominent dorsal macrotrichiae; *C*. *segnis* has a single faint macrotrichium in the second radial cell; *C*. *insignis* carries pigmentation along the extension of vein R3 [44, 45]. Furthermore, three species of Psychodidae that were identified: *B. olmeca olmeca*, *Micropygomyia cayennensis maciasi*, and *Psathyromyia* (*Forattiniella*) *texana* (Images and descriptions in material Supporting Information 5–7). Its identification was carried out following the morphological guidelines [46–48].

### 3.2. *Leishmania* spp. Infection Rates in Dipterans of Epidemiological Interest

Seven of 35 *C. furens* pools tested positive for *Leishmania* spp. DNA (Table 2), making this the most consequential molecular finding of the study. Positive results were spatially and temporally scattered: Tecualilla in July 2022, March 2023, and May 2023; Copales in September 2022 and March 2024; Ejido La Campana in September 2022; Palmillas in January 2023. No single locality was consistently positive across all rounds. Regarding incidence rates, La Campana recorded the highest values (125.0) and Copales the lowest (17.69). Twenty‐three specimens from the Psychodidae family were analyzed and tested negative for the presence of *Leishmania* DNA.

**Table 2:** PCR detection of *Leishmania* spp. DNA in *Culicoides furens* pools by locality and sampling date, with minimum infection rates (MIR).

#	Collection date	Tecualilla	Ej. La Campana	Palmillas	Copales
1	July 2022	**+**	−	−	−
2	Sep 2022	−	**+**	−	**+**
3	Nov 2022	−	−	0	−
4	Jan 2023	−	−	+	−
5	Mar 2023	**+**	−	−	−
6	May 2023	**+**	0	0	0
7	Jul 2023	0	0	0	−
8	Sep 2023	0	0	0	−
9	Nov 2023	0	−	0	−
10	Jan 2024	−	0	0	0
11	Mar 2024	−	−	0	**+**
12	Jun 2024	−	−	0	0
Females analyzed (n)		120	16	13	113
Pools positive		3	2	1	2
MIR per 1,000 individuals		25.0	125.0	76.9	17.7

*Note:* (+) positive; (–) negative; (0) no specimens collected. MIR = (positive pools/females analyzed) × 1000. Only blood‐feeding female *C. furens* were processed for molecular analysis. Pool sizes ranged from 1 to ~100 females per pool.

### 3.3. Seasonal Trophic Spectrum of Epidemiologically Relevant Dipterans

*C. furens* exhibited a markedly anthropophilic feeding pattern throughout the study area. Human DNA (*Homo sapiens*) was detected in pools from all four communities, with Tecualilla contributing the largest number of human‐positive pools (*n* = 12), followed by Ejido La Campana (*n* = 3), Palmillas (*n* = 2), and Copales (*n* = 2). In aggregate, human‐derived blood meals were identified in 19 of the pools in which host DNA was successfully amplified.

Dog DNA (*C. lupus familiaris*) was detected in a single pool, collected at Tecualilla during July 2023. No other host species were identified. The detection of *C. lupus familiaris* DNA in a pool from Tecualilla (the community with the highest overall peridomestic capture rate) is consistent with the known opportunistic feeding behavior of *C. furens* in environments where domestic animals co‐occur with humans at the forest edge.

*M. cayennensis maciasi* showed the contrasting pattern: dog DNA was detected in one pool from Copales (March 2024), whereas no human‐positive pools were recorded for this species. This peridomestic feeding event in *M. cayennensis maciasi* (a species whose preferred habitat is deep forest) carries separate epidemiological significance, as domestic dogs are the primary amplifying reservoir of *L. infantum* in urban and peridomestic transmission cycles across the Americas.

### 3.4. Association of Environmental Factors With Dipteran Abundance

Kruskal–Wallis tests detected significant habitat‐level differences in abundance for *C. furens* (*χ* ^2^(3) = 21.75, *p* < 0.001) and *M. cayennensis maciasi* (*χ* ^2^(3) = 13.34, *p* = 0.004) (Table 3). *C. furens* peaked at peripheral peridomestic backyards (Amb 1/1; median [IQR] = 3.75 [1.00–12.88]), whereas *M. cayennensis maciasi* was most abundant 300 m into the forest (Amb 2/2; median [IQR] = 0.25 [0.00–0.25]). The remaining species showed no significant habitat associations (*p* = 0.392 for all). Additionally, Fisher′s exact test applied to the three Psychodidae species across habitat types yielded nonsignificant results for all comparisons (*B. olmeca*: *p* = 0.185; *P. texana*: *p* = 1.000; *M. cayennensis*: *p* = 0.257), consistent with the Kruskal–Wallis findings and attributable to the extremely low specimen counts (1, 1, and 21 individuals, respectively).

**Table 3:** Median capture rates [IQR] by habitat and species. Kruskal–Wallis test (*χ* ^2^(3), df = 3). Asterisk ( ^∗^), statistically significant (*p* < 0.05). Amb 1/1: peripheral peridomestic backyard; Amb 1/2: central peridomestic backyard; Amb 2/1: 100 m into the forest; Amb 2/2: 300 m into the forest.

Species	Amb 1/1	Amb 1/2	Amb 2/1	Amb 2/2	KW *χ* ^2^(3)	*p*
*C. furens*	3.75 [1.00–12.88] ^∗^	0.00 [0.00–0.00]	0.88 [0.19–4.88]	0.88 [0.38–1.75]	21.75	< 0.001
*C. insignis*	0.00 [0.00–0.00]	0.00 [0.00–0.00]	0.00 [0.00–0.00]	0.00 [0.00–0.00]	ns	0.392
*C. segnis*	0.00 [0.00–0.00]	0.00 [0.00–0.00]	0.00 [0.00–0.00]	0.00 [0.00–0.00]	ns	0.392
*B. olmeca*	0.00 [0.00–0.00]	0.00 [0.00–0.00]	0.00 [0.00–0.00]	0.00 [0.00–0.00]	ns	0.392
*P. texana*	0.00 [0.00–0.00]	0.00 [0.00–0.00]	0.00 [0.00–0.00]	0.00 [0.00–0.00]	ns	0.392
*M. cayennensis*	0.00 [0.00–0.25]	0.00 [0.00–0.00]	0.00 [0.00–0.00]	0.25 [0.00–0.25] ^∗^	13.34	0.004

Abbreviations: IQR, interquartile range (Q1–Q3); ns, not significant.

^∗^ *p* < 0.05.

**Figure 3:**
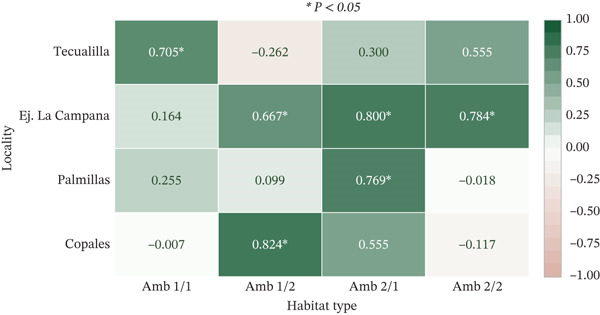
Spearman rho heat map between rainfall and dipteran abundance by locality and habitat. Bold values with asterisk (∗) = *p* < 0.05. Green = positive; red = negative correlation.

Spearman correlations between rainfall and dipteran abundance were positive and often significant across localities and habitat types. Notable values included rs = 0.705 (Tecualilla, Amb 1/1, *p* < 0.05), rs = 0.824 (Copales, Amb 1/2, *p* < 0.01), and rs = 0.800 (Ejido La Campana, Amb 2/1, *p* < 0.01). Wind speed was inversely correlated with abundance in Tecualilla forest sites (rs = −0.712, Amb 2/1, *p* < 0.01). These patterns are illustrated in the correlation heat map (Figure 3).

## 4. Discussion

### 4.1. Spatial and Seasonal Patterns of Dipteran Abundance

Our collection of 9984 dipterans across 12 sampling rounds in four southern Sinaloan communities confirmed what we had suspected going into the study: forest environments are the principal reservoir of vector populations in this coastal landscape. Capturing 81.5% of specimens in sylvatic sites, versus less than 20% in village backyards, mirrors findings from Yucatan, where forest conservation is positively associated with sand fly richness and abundance [27, 51]. The structural complexity of forest soil, its moisture retention, and the availability of decomposing organic matter all favor ceratopogonid and phlebotomine reproduction in ways that village environments simply cannot match [51].

Seasonal peaks in September and November, with minima between May and July, reflect the strongly seasonal rainfall in Escuinapa. The same rhythm has been documented for *Culicoides paraensis* in the Brazilian Amazon, where ceratopogonid populations were roughly three times higher during the wet season than the dry [52]. Long‐term monitoring in the United Kingdom found that rising precipitation and temperature together extended the *Culicoides* active season by up to 40 days [53], a finding with direct implications for disease risk as climate shifts. If projected increases in temperature and precipitation extend the seasonal activity period of *Culicoides* and phlebotomine sand fly populations in northwestern Mexico, the risk of human exposure at the forest‐peridomestic interface is expected to intensify accordingly.

### 4.2. Species Composition and Vector Relevance

*C. furens* dominated the Ceratopogonidae (mean 33.09; *p* < 0.05), a result consistent with its broad distribution in coastal tropical habitats throughout the Americas. Spinelli and Wolff [54] recorded it among new species for the Sinaloa coast, and its presence in both peridomestic and forested sites (where breeding substrates are abundant) was predictable from the literature. Beyond its nuisance value, *C. furens* has been implicated as a carrier of filariae [55], and was associated with *L. mexicana* DNA in an earlier survey from the same region [30].

Among the Psychodidae, the collection of *B. olmeca olmeca* deserves particular attention. This is the only sand fly with confirmed vector status for *L. mexicana* in Mexico, with documented anthropophily [33, 56]. Lozano‐Sardaneta et al. [57] recently updated its national distribution, noting that suitable habitat follows patterns of wet‐season rainfall and cold‐month temperature climatic conditions that broadly characterize southern Sinaloa. Recording this species here, to our knowledge, is the first documented occurrence in Sinaloa and constitutes a find of genuine epidemiological weight for the northwest of Mexico.

### 4.3. *Leishmania* spp. Detection in *C. furens*

Detecting *Leishmania* spp. DNA in 7 of 35 *C. furens* pools was the most striking finding we obtained. Rebêlo et al. [58] were among the first to report *Leishmania* DNA (specifically *L. braziliensis* and *L. amazonensis*) in multiple *Culicoides* species in the Brazilian Amazon, concluding that natural contact with infected hosts was demonstrable even if vector competence remained unproven. Ríos‐Tostado et al. [30] did the same for *C. furens* in northwestern Mexico, making their study the closest geographic antecedent to what we found here.

It is essential to clarify the scope and limitations of our PCR findings. Molecular detection in pools of *C*. *furens* should be interpreted as evidence of contact with *Leishmania* infected hosts, not as demonstration of vectorial capacity, since amplification of parasite DNA from whole‐insect homogenates does not differentiate digested parasites from viable, replication‐competent infections. Seblová et al. [59] showed experimentally that *Culicoides sonorensis* can harbour late‐stage *Leishmania enrietti* up to the stomodeal valve, which is the right anatomical position for transmission during a subsequent bite. Studies from Thailand implicated *Culicoides peregrinus* as a possible vector of *L. martiniquensis* via microscopic identification of promastigotes in the foregut [60, 61], though those parasites belong to the subgenus *Mundinia*, phylogenetically distant from *L. mexicana*. We therefore treat the data as evidence that *C. furens* in Escuinapa feeds on *Leishmania*‐infected vertebrates, but experimental infection studies are needed before we can draw conclusions about transmission.

### 4.4. Infection Rates in Psychodidae

All 23 sand fly specimens tested negative for *Leishmania* DNA. The lack of positive findings is most plausibly attributed to the relatively small sample size examined. Natural infection prevalences in phlebotomines typically range from 0% to 83% in endemic areas of southeastern Mexico. In samples of thousands of specimens, these values vary according to the month of collection [56]. To reliably detect the infection at that prevalence requires several hundred specimens at minimum. Capturing only 23 sand flies over 2 years reflects the genuinely sparse populations in the area, possibly because we are near the northern distributional limits of these species, or because local soil moisture and temperature profiles fall below the thresholds that favor larval development [57, 62]. The negative result should not be read as evidence against *Leishmania* circulation in sand flies here.

### 4.5. Trophic Spectrum

*C. furens* fed predominantly on humans: Tecualilla alone yielded 12 pools positive for human DNA, and at least one human‐positive pool was recorded at each locality. Dog DNA (*C. lupus familiaris*) was detected in only one pool, in Tecualilla in July 2023. This strong anthropophilic signal is consistent with reports from Thailand, where *Culicoides* spp. primarily targeting cattle also opportunistically bite humans when preferred hosts are absent [63]. The combination of *Leishmania* DNA detection and anthropophily in the same species is what makes *C. furens* the most epidemiologically concerning taxon in our study area.

*B. olmeca* fed on humans at Tecualilla (two pools) and Ejido La Campana (one pool). Shaw [64] recent review notes that whereas *B. olmeca* has a preference for wild rodents, it is attracted to humans under certain conditions (particularly where forest disturbance reduces rodent density) a scenario that fits Escuinapa′s mosaic landscape well. The detection of *C. lupus familiaris* DNA in a *M. cayennensis maciasi* pool from Copales (March 2024) carries separate epidemiological weight. Domestic dogs are the main amplifying reservoir of *L. infantum* in urban and peridomestic transmission cycles in the Americas, with cutaneous parasite loads high enough to sustain infection in feeding sand flies [65]. Dantas‐Torres [66] has mapped canine leishmaniasis across the continent and stressed that infected dogs in peridomestic settings substantially elevate transmission risk when anthropophilic vectors co‐occur. Critically, no human‐positive pools were recorded for *M*. *cayennensis maciasi* in this study; this species therefore defines a peridomestic risk pathway through the canine reservoir cycle rather than a direct human exposure interface. The feeding event at Copales nonetheless warrants dedicated follow‐up to determine its potential role in the peridomestic transmission cycle.

### 4.6. Environmental Correlates of Abundance

Rainfall emerged as the single most consistent environmental driver of dipteran abundance, with significant positive correlations in both peridomestic and forest environments across multiple localities. The effect operated through an increase in breeding site availability consistent with the observation by García‐Suárez et al. [67] that accumulated precipitation 4 weeks before sampling was the dominant predictor of mosquito abundance in Yucatan, and with the Amazonian data of Maciel‐Feitoza et al. [52] showing that *C. paraensis* populations track the rainy season closely. Highest correlation coefficients came from central village backyards (Copales, rs = 0.824, *p* < 0.01) and deep forest sites (Ejido La Campana, *r* = 0.800, *p* < 0.01), both of which retain moisture long after rainfall events. The negative correlation between wind speed and abundance in Tecualilla forest sites (*r* = −0.712, *p* < 0.01) makes ecological sense: ceratopogonids have a size < 2 mm with limited powered flight, and winds above approximately 2 m/s disrupt both their flight and biting activity, as well as their ability to capture prey in traps. [68, 69]. Rainfall was the only climatic variable with significant, consistent positive correlations across localities and habitat types. Temperature, relative humidity, and wind speed showed nonsignificant or inconsistent patterns (full data available in Supporting Information 8), justifying the emphasis on precipitation in Figure 3. The contrasting habitat preferences of the two epidemiologically relevant dipterans (*C. furens* concentrated at the peridomestic periphery and *M. cayennensis* deepest in the forest) define two distinct risk interfaces. The peridomestic edge is accessible to villagers going about daily life; it is also the interface that control interventions can most readily target. The 300 m forest zone represents a more cryptic exposure pathway, primarily relevant for people who enter the forest for work or recreation. Montes de Oca‐Aguilar et al. [51] showed that landscape heterogeneity at the forest‐community edge elevates the presence of anthropophilic sand flies close to inhabited areas, meaning that forest fragmentation, which is ongoing in the municipality of Escuinapa and throughout the southern part of the state, may worsen the peridomestic exposure risk without any need for people to enter the forest at all.

### 4.7. Limitations

The following limitations should be considered when interpreting our findings. First, only 23 phlebotomine sand fly specimens were collected over 2 years, a sample far too small to reliably detect natural *Leishmania* infection prevalences, which typically range from 0% to 83% in endemic areas and require several hundred specimens for adequate statistical power; the absence of *Leishmania* DNA in Psychodidae should therefore not be interpreted as evidence against sand fly involvement in transmission. Second, positive PCR products from *C. furens* pools were not subjected to confirmatory sequencing, so *Leishmania* detections are reported at the genus level (*Leishmania* spp.) rather than species level throughout this manuscript. Third, CDC light traps were operated during the full‐moon phase, which may reduce the activity of photoaversive phlebotomine species and could have contributed to the low Psychodidae capture rates. Fourth, captures were not standardized beyond protocol uniformity, which may limit quantitative comparisons across localities with different vegetation densities. Fifth, the observational design of this study precludes causal inference regarding vector competence; experimental infection studies with *C. furens* and *Leishmania* spp. remain an essential next step.

## 5. Conclusions

Our 2‐year survey establishes the forest–community interface of southern Sinaloa as an active contact zone between human populations and dipterans capable of harboring *Leishmania*. Forest environments concentrated 81.5% of captures, and abundance peaks in September–November tracked seasonal rainfall. *C. furens* was the dominant ceratopogonid, was found feeding on humans across all four communities, and carried *Leishmania* spp. DNA in 7 of 35 pools tested together making it the species of greatest immediate epidemiological concern. Critically, however, molecular detection of parasite DNA in a blood‐sucking insect is not a demonstration of vector competence; experimental infection studies remain an essential next step. The first documentation of *B. olmeca olmeca* in Sinaloa is significant not due to species abundance, which remained low, but rather because it represents the sole sand fly species with confirmed vector competence for *L. mexicana* in Mexico, and we documented its anthropophilic feeding behavior here. The failure to detect *Leishmania* in sand flies is almost certainly a statistical artifact of the small sample size (*n* = 23), not evidence that sand flies are uninvolved in transmission. The niche differentiation we observed (*C. furens* at the village edge, *M. cayennensis* deep in the forest) points to separate peridomestic and sylvatic exposure pathways that could correspond to distinct putative transmission dynamics. As climate projections for northwestern Mexico trend toward warmer temperatures and altered precipitation regimes, both interfaces are likely to see longer transmission seasons. We believe that future studies should focus on experimentally evaluating the vector capacity of *C*. *furens*, conducting epidemiological surveillance of sand flies with larger numbers, and determining the relationship between these flies and the sylvatic and peridomestic infection cycles.

## Funding

No funding was received for this manuscript.

## Conflicts of Interest

The authors declare no conflicts of interest.

## Supporting Information

Additional supporting information can be found online in the Supporting Information section.

## Supporting information


**Supporting Information 1.** Traps location.

**Supporting Information 2.** Statistical workflow.

**Supporting Information 3.** Number of diptera collected per trap type, locality, and sampling period.

**Supporting Information 4.** Morphological features of Ceratopogonidae.

**Supporting Information 5.** Female of *Bichormomyia olmeca olmeca.*

**Supporting Information 6.** Female of *Micropygomyia cayennensis maciasi.*

**Supporting Information 7.** Male of *Psathyromyia* (*Foratinella*) *texana.*

**Supporting Information 8.** Climatic variables recorded at each sampling.

## Data Availability

The data that support the findings of this study are available from the corresponding author upon reasonable request.
