# Supplementary material for: Leishmaniasis Transmission Risk at the Forest‐Peridomestic Interface in an Area of Southern Sinaloa, Mexico: Entomological, Molecular, and Climatic Evidence
Source: J Parasitol Res. 2026 Jun 16;2026:5071505. doi: 10.1155/japr/5071505 (PMC13270774; doi:10.1155/japr/5071505)
Supplement: Supplementary file 1 — Supporting Information 1. Traps location. [file JAPR-2026-5071505-s007.pptx]

## Slide 1
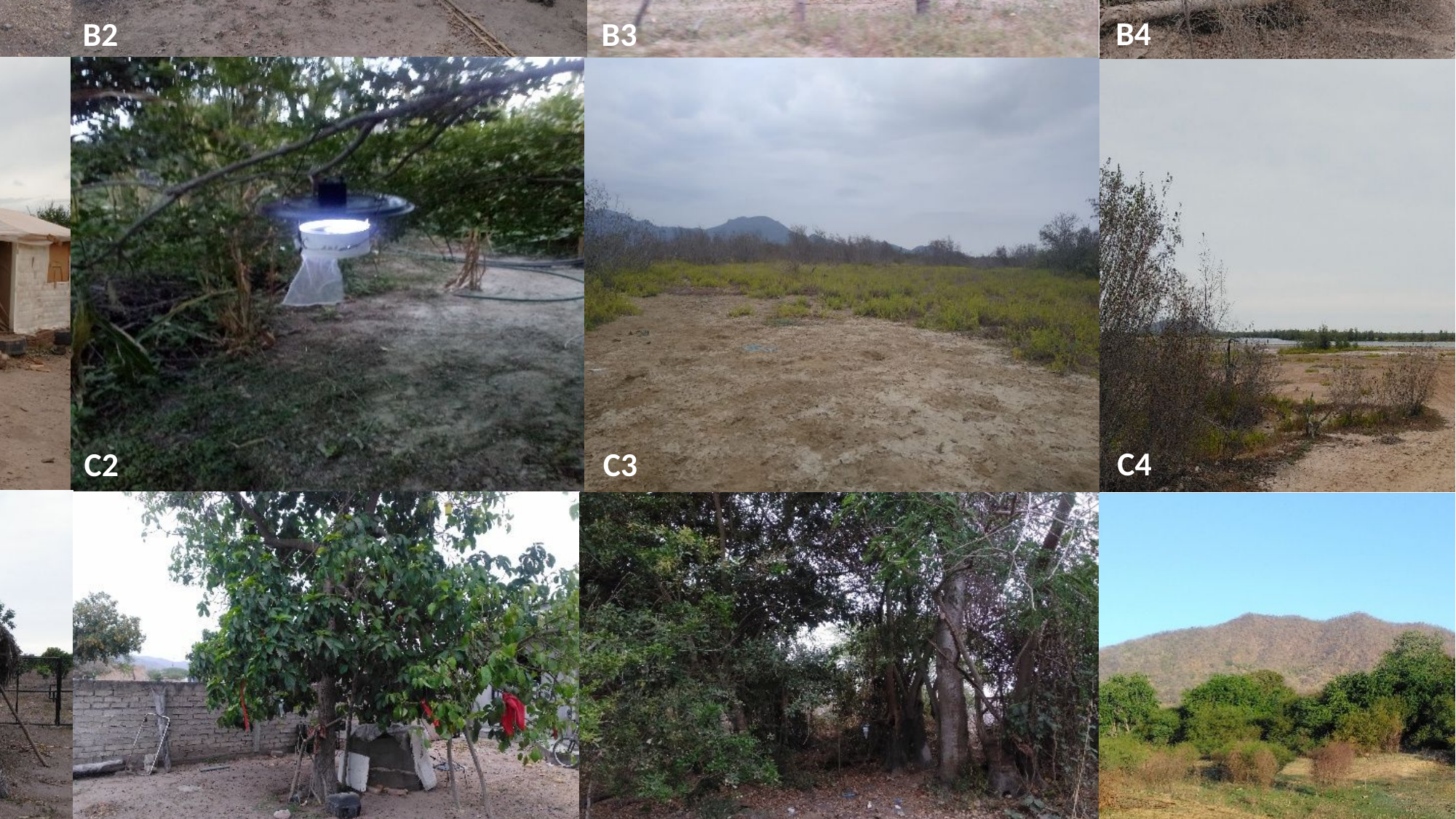

A4
A3
A2
A1
B4
B3
B2
B1
C4
C3
C2
C1
D4
D3
D2
D1

## Slide 2
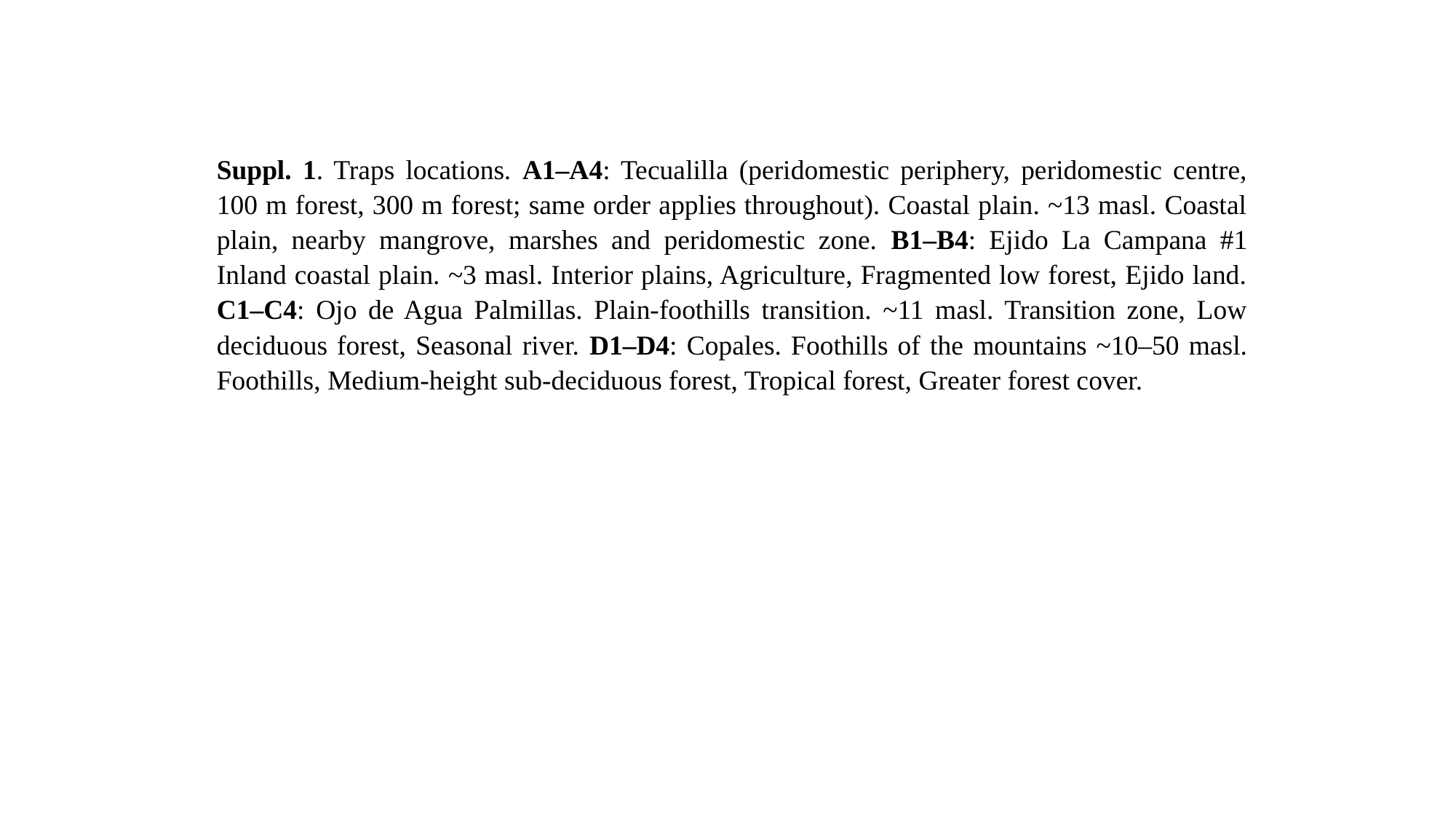

Suppl. 1. Traps locations. A1–A4: Tecualilla (peridomestic periphery, peridomestic centre, 100 m forest, 300 m forest; same order applies throughout). Coastal plain. ~13 masl. Coastal plain, nearby mangrove, marshes and peridomestic zone. B1–B4: Ejido La Campana #1 Inland coastal plain. ~3 masl. Interior plains, Agriculture, Fragmented low forest, Ejido land. C1–C4: Ojo de Agua Palmillas. Plain-foothills transition. ~11 masl. Transition zone, Low deciduous forest, Seasonal river. D1–D4: Copales. Foothills of the mountains ~10–50 masl. Foothills, Medium-height sub-deciduous forest, Tropical forest, Greater forest cover.
