# Supplementary material for: Leishmaniasis Transmission Risk at the Forest‐Peridomestic Interface in an Area of Southern Sinaloa, Mexico: Entomological, Molecular, and Climatic Evidence
Source: J Parasitol Res. 2026 Jun 16;2026:5071505. doi: 10.1155/japr/5071505 (PMC13270774; doi:10.1155/japr/5071505)
Supplement: Supplementary file 2 — Supporting Information 2. Statistical workflow. [file JAPR-2026-5071505-s001.pptx]

## Slide 1
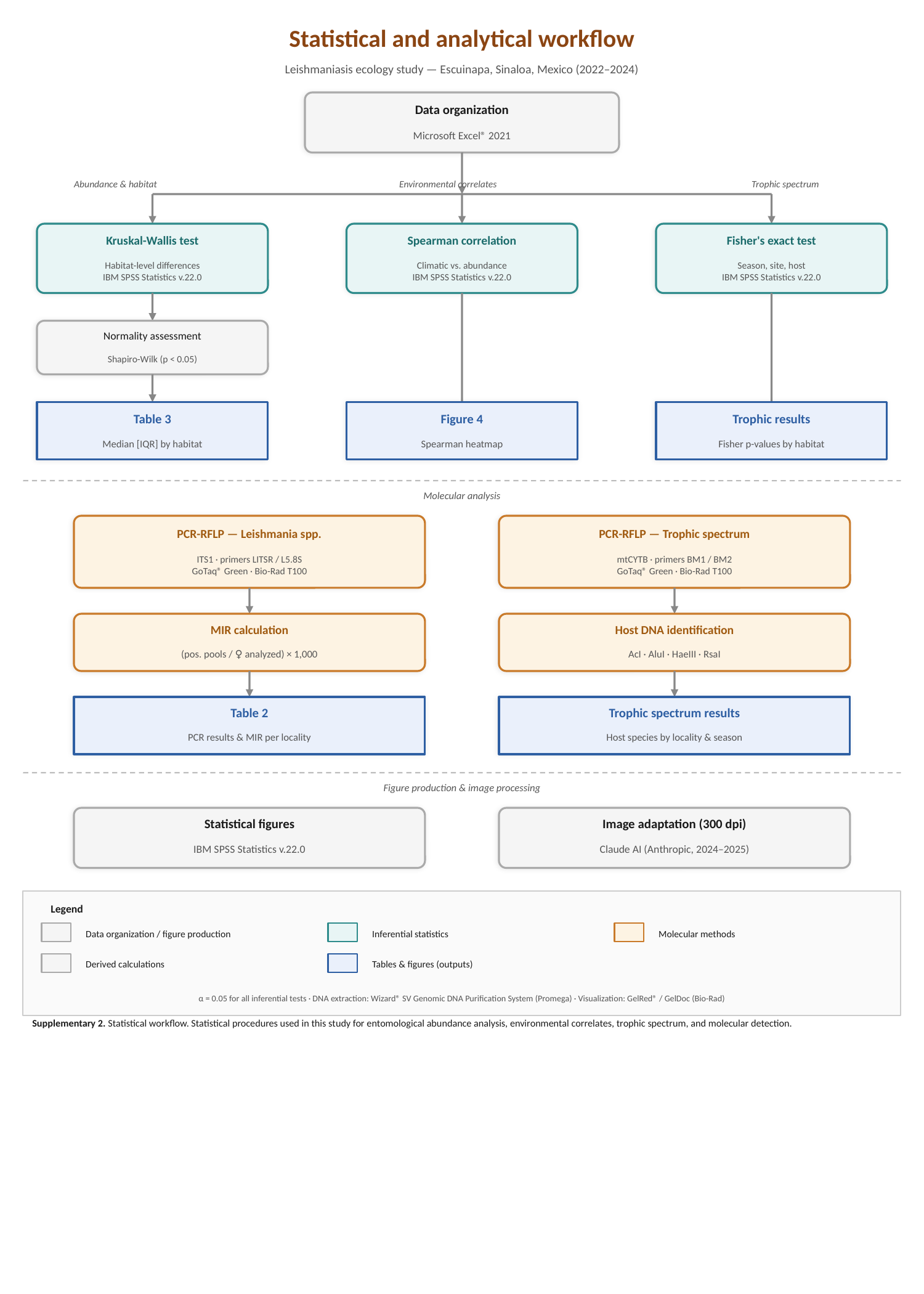

Statistical and analytical workflow
Leishmaniasis ecology study — Escuinapa, Sinaloa, Mexico (2022–2024)
Data organization
Microsoft Excel® 2021
Abundance & habitat
Environmental correlates
Trophic spectrum
Kruskal-Wallis test
Spearman correlation
Fisher's exact test
Habitat-level differences
IBM SPSS Statistics v.22.0
Climatic vs. abundance
IBM SPSS Statistics v.22.0
Season, site, host
IBM SPSS Statistics v.22.0
Normality assessment
Shapiro-Wilk (p < 0.05)
Table 3
Figure 4
Trophic results
Median [IQR] by habitat
Spearman heatmap
Fisher p-values by habitat
Molecular analysis
PCR-RFLP — Leishmania spp.
PCR-RFLP — Trophic spectrum
ITS1 · primers LITSR / L5.8S
GoTaq® Green · Bio-Rad T100
mtCYTB · primers BM1 / BM2
GoTaq® Green · Bio-Rad T100
MIR calculation
Host DNA identification
(pos. pools / ♀ analyzed) × 1,000
AcI · AluI · HaeIII · RsaI
Table 2
Trophic spectrum results
PCR results & MIR per locality
Host species by locality & season
Figure production & image processing
Statistical figures
Image adaptation (300 dpi)
IBM SPSS Statistics v.22.0
Claude AI (Anthropic, 2024–2025)
Legend
Data organization / figure production
Inferential statistics
Molecular methods
Derived calculations
Tables & figures (outputs)
α = 0.05 for all inferential tests · DNA extraction: Wizard® SV Genomic DNA Purification System (Promega) · Visualization: GelRed® / GelDoc (Bio-Rad)
Supplementary 2. Statistical workflow. Statistical procedures used in this study for entomological abundance analysis, environmental correlates, trophic spectrum, and molecular detection.
