# Supplementary material for: Leishmaniasis Transmission Risk at the Forest‐Peridomestic Interface in an Area of Southern Sinaloa, Mexico: Entomological, Molecular, and Climatic Evidence
Source: J Parasitol Res. 2026 Jun 16;2026:5071505. doi: 10.1155/japr/5071505 (PMC13270774; doi:10.1155/japr/5071505)
Supplement: Supplementary file 3 — Supporting Information 3. Number of diptera collected per trap type, locality, and sampling period. [file JAPR-2026-5071505-s002.docx]

**Supplementary 3.**

Number of Diptera collected per trap type, locality, and sampling period (July 2023 – June 2025). Trap codes: TP01 = peridomestic, town center; TP02 = peridomestic, town outskirts; TB01 = sylvatic, 100 m into forest; TB02 = sylvatic, 300 m into forest.

|  |  | **Sampling period** | | | | | | | | | | | |  |
| --- | --- | --- | --- | --- | --- | --- | --- | --- | --- | --- | --- | --- | --- | --- |
| **Locality** | **Trap** | **Jul 2023** | **Sep 2023** | **Nov 2023** | **Jan 2024** | **Mar 2024** | **May 2024** | **Jul 2024** | **Sep 2024** | **Nov 2024** | **Jan 2025** | **Mar 2025** | **Jun 2025** | **Total** |
| **Tecualilla** | | | | | | | | | | | | | | |
|  | TP01 | 195 | 119 | 95 | 8 | 15 | 53 | 15 | 89 | 19 | 4 | 14 | 5 | **631** |
|  | TP02 | 23 | 8 | 2 | 20 | 7 | 18 | 6 | 4 | 32 | 9 | 24 | 1 | **154** |
|  | TB01 | 9 | 207 | 192 | 213 | 183 | 0 | 44 | 222 | 210 | 39 | 124 | 46 | **1489** |
|  | TB02 | 97 | 681 | 18 | 24 | 24 | 0 | 45 | 109 | 90 | 9 | 50 | 14 | **1161** |
|  | **Subtotal** | **324** | **1015** | **307** | **265** | **229** | **71** | **110** | **424** | **351** | **61** | **212** | **66** | **3435** |
| **Ej. La Campana** | | | | | | | | | | | | | | |
|  | TP01 | 3 | 3 | 2 | 0 | 13 | 0 | 8 | 33 | 65 | 7 | 9 | 4 | **147** |
|  | TP02 | 4 | 14 | 0 | 6 | 38 | 0 | 3 | 61 | 5 | 9 | 10 | 3 | **153** |
|  | TB01 | 11 | 34 | 24 | 6 | 4 | 1 | 30 | 196 | 54 | 5 | 34 | 8 | **407** |
|  | TB02 | 78 | 57 | 6 | 18 | 15 | 5 | 37 | 160 | 107 | 7 | 15 | 2 | **507** |
|  | **Subtotal** | **96** | **108** | **32** | **30** | **70** | **6** | **78** | **450** | **231** | **28** | **68** | **17** | **1214** |
| **Palmillas** | | | | | | | | | | | | | | |
|  | TP01 | 3 | 6 | 1 | 10 | 0 | 2 | 3 | 30 | 9 | 6 | 4 | 2 | **76** |
|  | TP02 | 3 | 4 | 0 | 14 | 0 | 0 | 14 | 4 | 7 | 0 | 0 | 0 | **46** |
|  | TB01 | 8 | 9 | 1 | 34 | 1 | 1 | 42 | 180 | 3 | 7 | 6 | 24 | **316** |
|  | TB02 | 2 | 113 | 869 | 7 | 10 | 5 | 5 | 113 | 41 | 6 | 9 | 2 | **1182** |
|  | **Subtotal** | **16** | **132** | **871** | **65** | **11** | **8** | **64** | **327** | **60** | **19** | **19** | **28** | **1620** |
| **Copales** | | | | | | | | | | | | | | |
|  | TP01 | 3 | 5 | 194 | 47 | 107 | 0 | 2 | 19 | 29 | 0 | 22 | 0 | **428** |
|  | TP02 | 7 | 38 | 1 | 6 | 4 | 0 | 14 | 90 | 46 | 8 | 1 | 0 | **215** |
|  | TB01 | 9 | 9 | 154 | 27 | 119 | 1 | 29 | 289 | 62 | 5 | 20 | 2 | **726** |
|  | TB02 | 35 | 163 | 260 | 900 | 96 | 1 | 72 | 157 | 311 | 33 | 267 | 51 | **2346** |
|  | **Subtotal** | **54** | **215** | **609** | **980** | **326** | **2** | **117** | **555** | **448** | **46** | **310** | **53** | **3715** |
| **Grand total** |  | **490** | **1470** | **1819** | **1340** | **636** | **87** | **369** | **1756** | **1090** | **154** | **609** | **164** | **9984** |

TP01 = Peridomestic trap, town center; TP02 = Peridomestic trap, town outskirts; TB01 = Sylvatic trap, 100 m into forest; TB02 = Sylvatic trap, 300 m into forest.

Sampling was conducted bimonthly from July 2023 to June 2025 across four localities in the study area.
