# Supplementary material for: Leishmaniasis Transmission Risk at the Forest‐Peridomestic Interface in an Area of Southern Sinaloa, Mexico: Entomological, Molecular, and Climatic Evidence
Source: J Parasitol Res. 2026 Jun 16;2026:5071505. doi: 10.1155/japr/5071505 (PMC13270774; doi:10.1155/japr/5071505)
Supplement: Supplementary file 4 — Supporting Information 4. Morphological features of Ceratopogonidae. [file JAPR-2026-5071505-s008.pptx]

## Slide 1
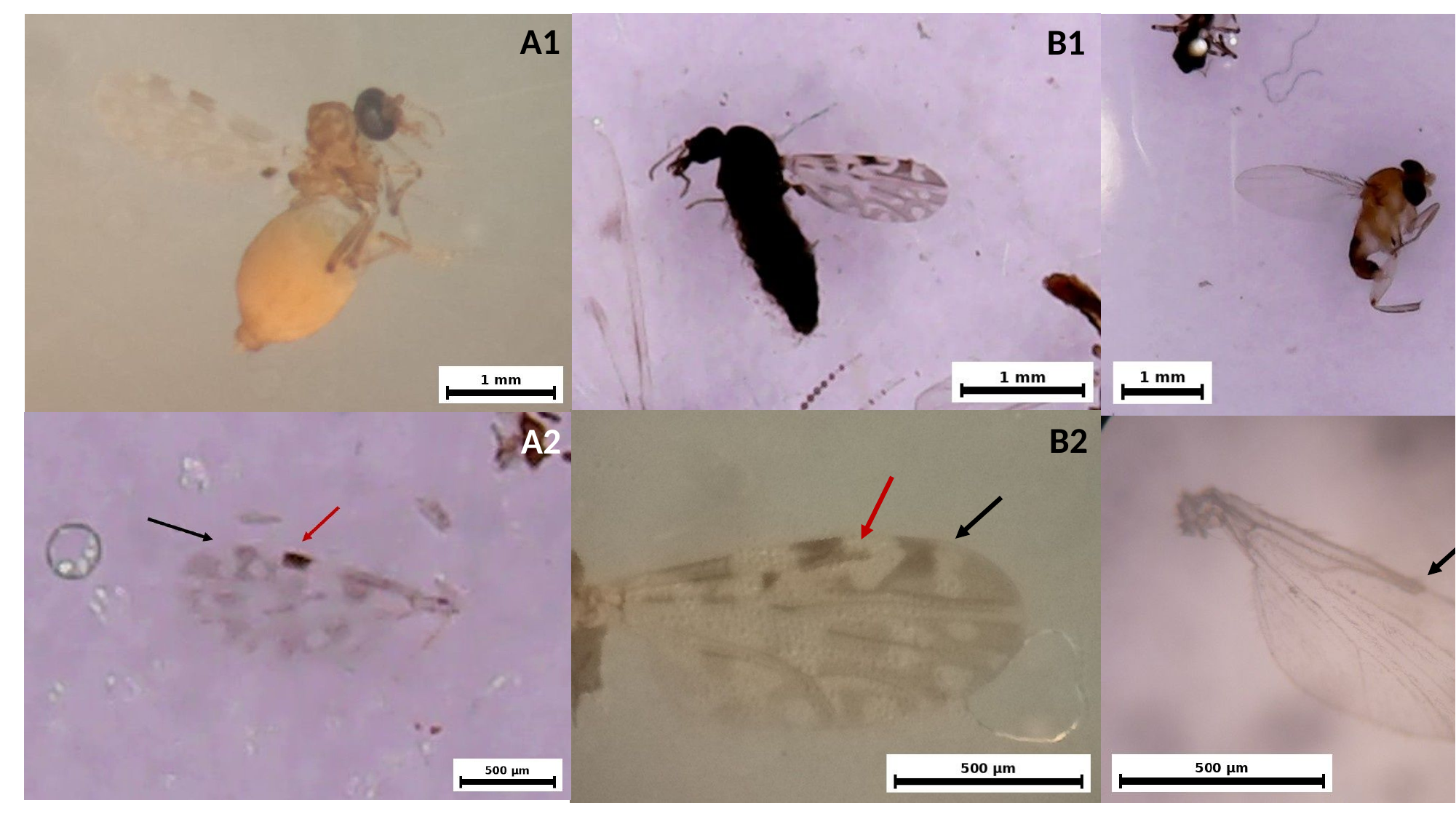

B1
A1
B1
C1
B2
B2
A2
A2
C2

## Slide 2
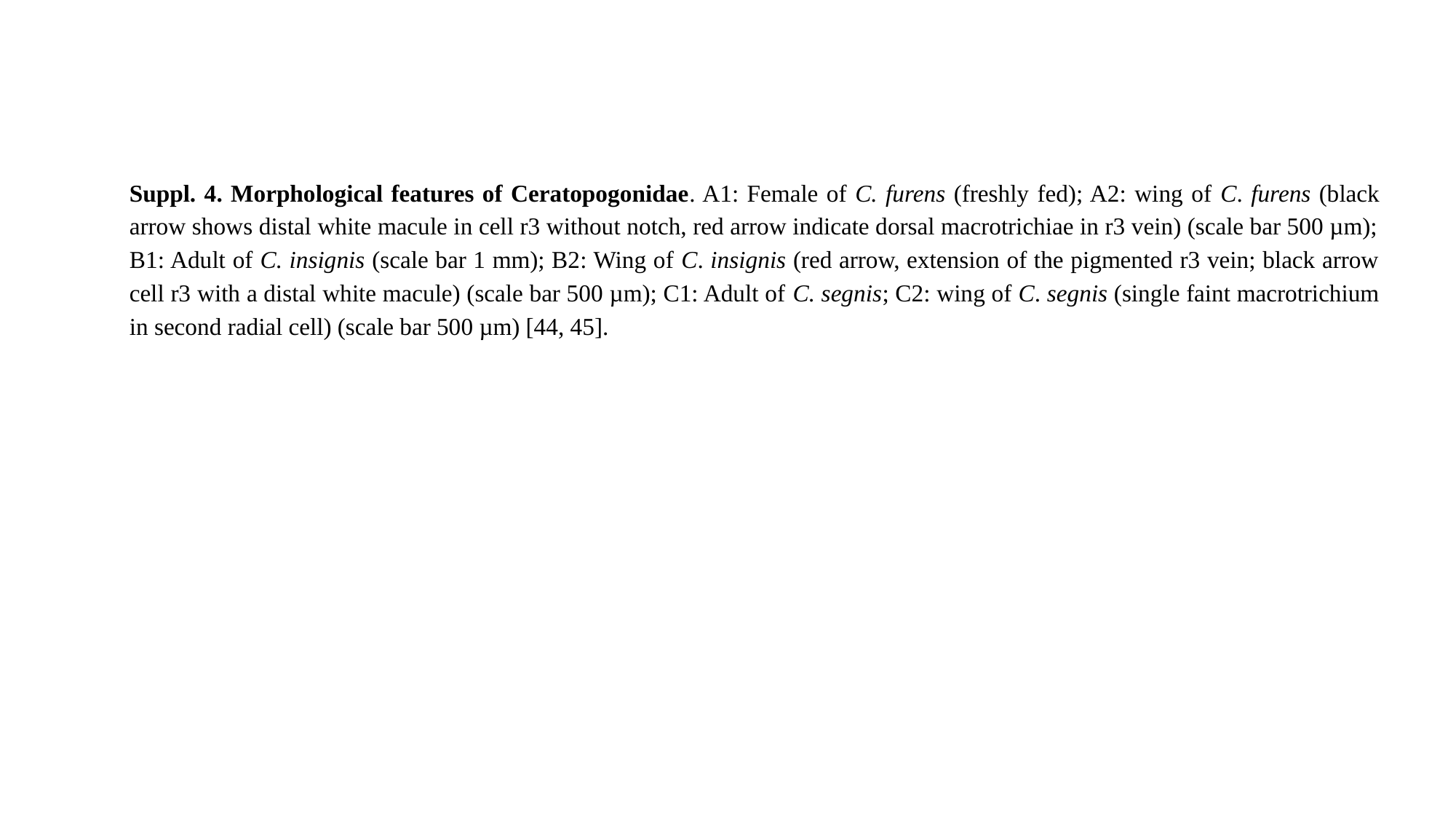

Suppl. 4. Morphological features of Ceratopogonidae. A1: Female of C. furens (freshly fed); A2: wing of C. furens (black arrow shows distal white macule in cell r3 without notch, red arrow indicate dorsal macrotrichiae in r3 vein) (scale bar 500 µm); B1: Adult of C. insignis (scale bar 1 mm); B2: Wing of C. insignis (red arrow, extension of the pigmented r3 vein; black arrow cell r3 with a distal white macule) (scale bar 500 µm); C1: Adult of C. segnis; C2: wing of C. segnis (single faint macrotrichium in second radial cell) (scale bar 500 µm) [44, 45].
