# Supplementary material for: Leishmaniasis Transmission Risk at the Forest‐Peridomestic Interface in an Area of Southern Sinaloa, Mexico: Entomological, Molecular, and Climatic Evidence
Source: J Parasitol Res. 2026 Jun 16;2026:5071505. doi: 10.1155/japr/5071505 (PMC13270774; doi:10.1155/japr/5071505)
Supplement: Supplementary file 5 — Supporting Information 5. Female of Bichormomyia olmeca olmeca. [file JAPR-2026-5071505-s004.pptx]

## Slide 1
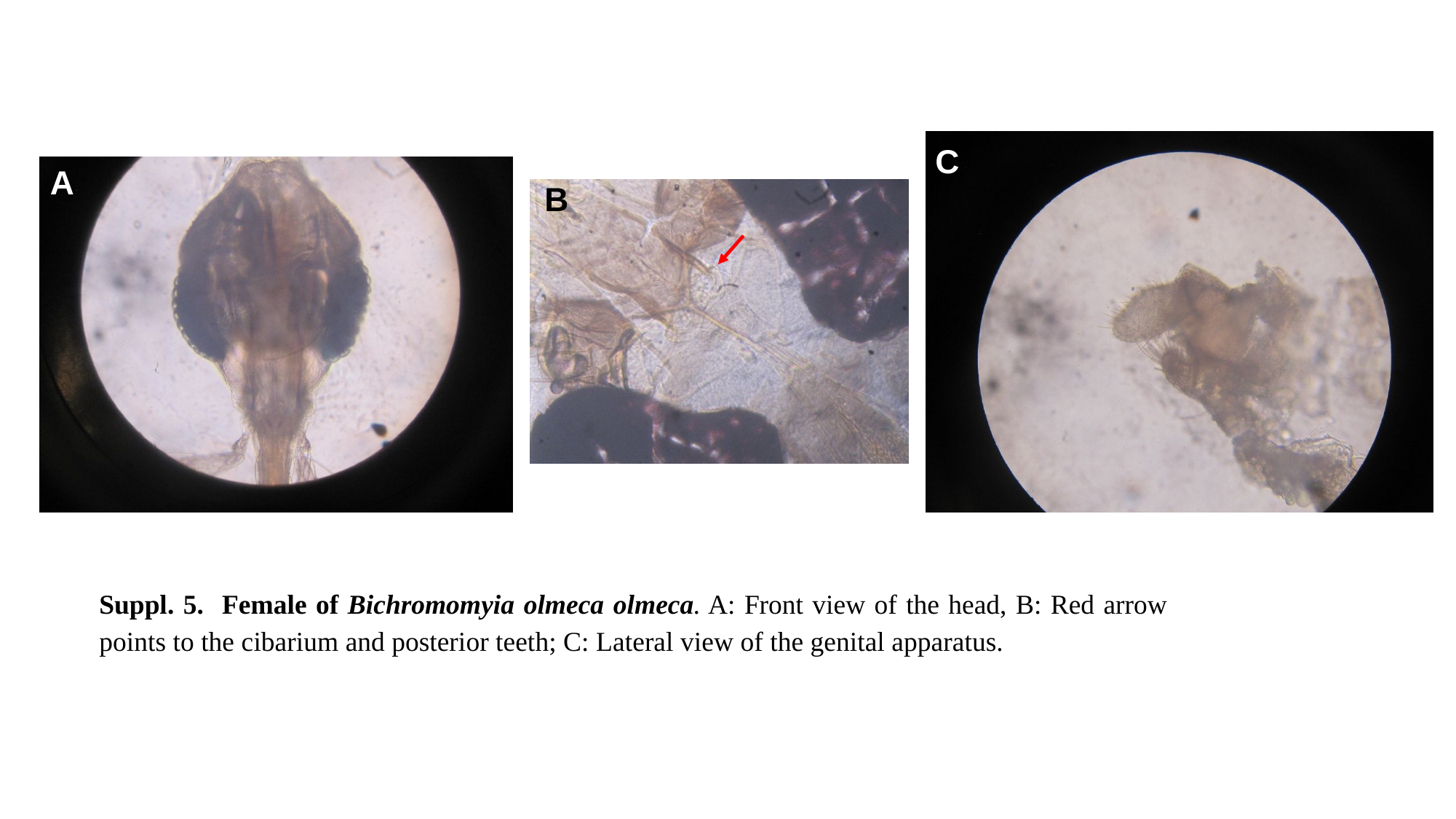

C
A
B
Suppl. 5. Female of Bichromomyia olmeca olmeca. A: Front view of the head, B: Red arrow points to the cibarium and posterior teeth; C: Lateral view of the genital apparatus.
