# Supplementary material for: Leishmaniasis Transmission Risk at the Forest‐Peridomestic Interface in an Area of Southern Sinaloa, Mexico: Entomological, Molecular, and Climatic Evidence
Source: J Parasitol Res. 2026 Jun 16;2026:5071505. doi: 10.1155/japr/5071505 (PMC13270774; doi:10.1155/japr/5071505)
Supplement: Supplementary file 6 — Supporting Information 6. Female of Micropygomyia cayennensis maciasi. [file JAPR-2026-5071505-s005.pptx]

## Slide 1
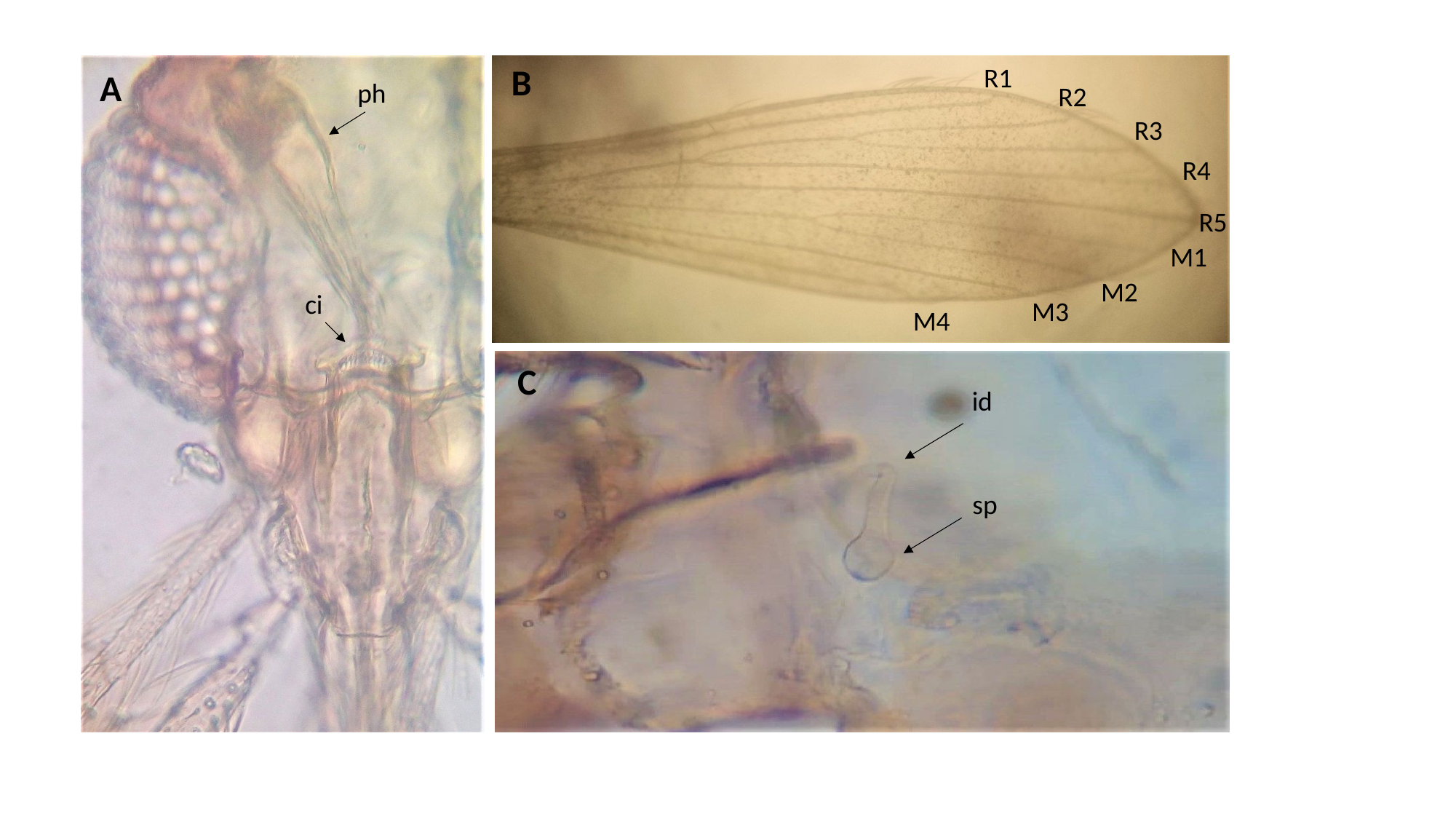

B
R1
A
ph
R2
R3
R4
R5
M1
M2
ci
M3
M4
C
id
sp

## Slide 2
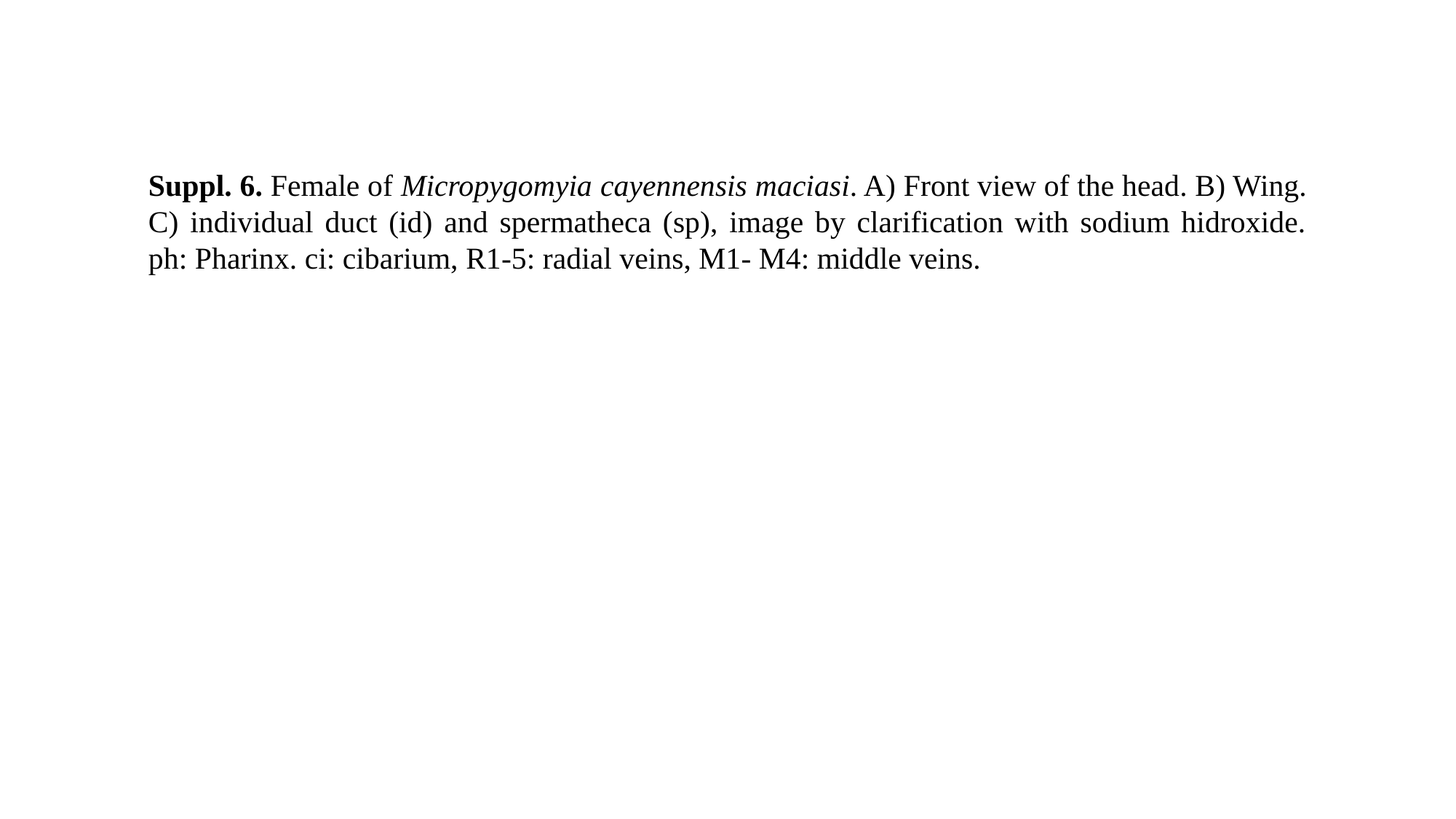

Suppl. 6. Female of Micropygomyia cayennensis maciasi. A) Front view of the head. B) Wing. C) individual duct (id) and spermatheca (sp), image by clarification with sodium hidroxide. ph: Pharinx. ci: cibarium, R1-5: radial veins, M1- M4: middle veins.
