# Supplementary material for: Leishmaniasis Transmission Risk at the Forest‐Peridomestic Interface in an Area of Southern Sinaloa, Mexico: Entomological, Molecular, and Climatic Evidence
Source: J Parasitol Res. 2026 Jun 16;2026:5071505. doi: 10.1155/japr/5071505 (PMC13270774; doi:10.1155/japr/5071505)
Supplement: Supplementary file 7 — Supporting Information 7. Male of Psathyromyia (Foratinella) texana. [file JAPR-2026-5071505-s006.pptx]

## Slide 1
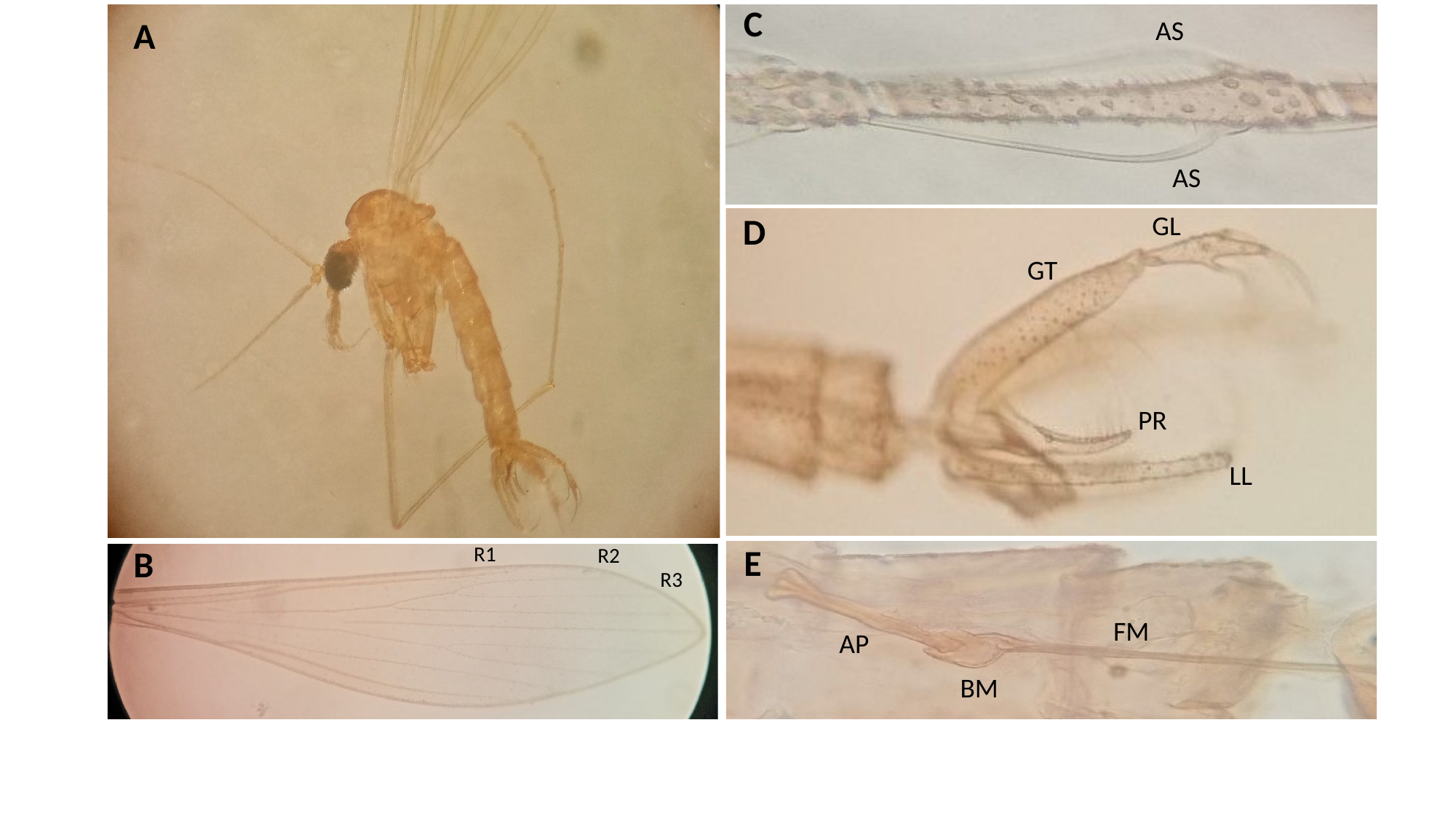

C
A
AS
R1
B
R2
AS
GL
GT
PR
LL
D
E
R1
B
R2
R3
FM
AP
BM

## Slide 2
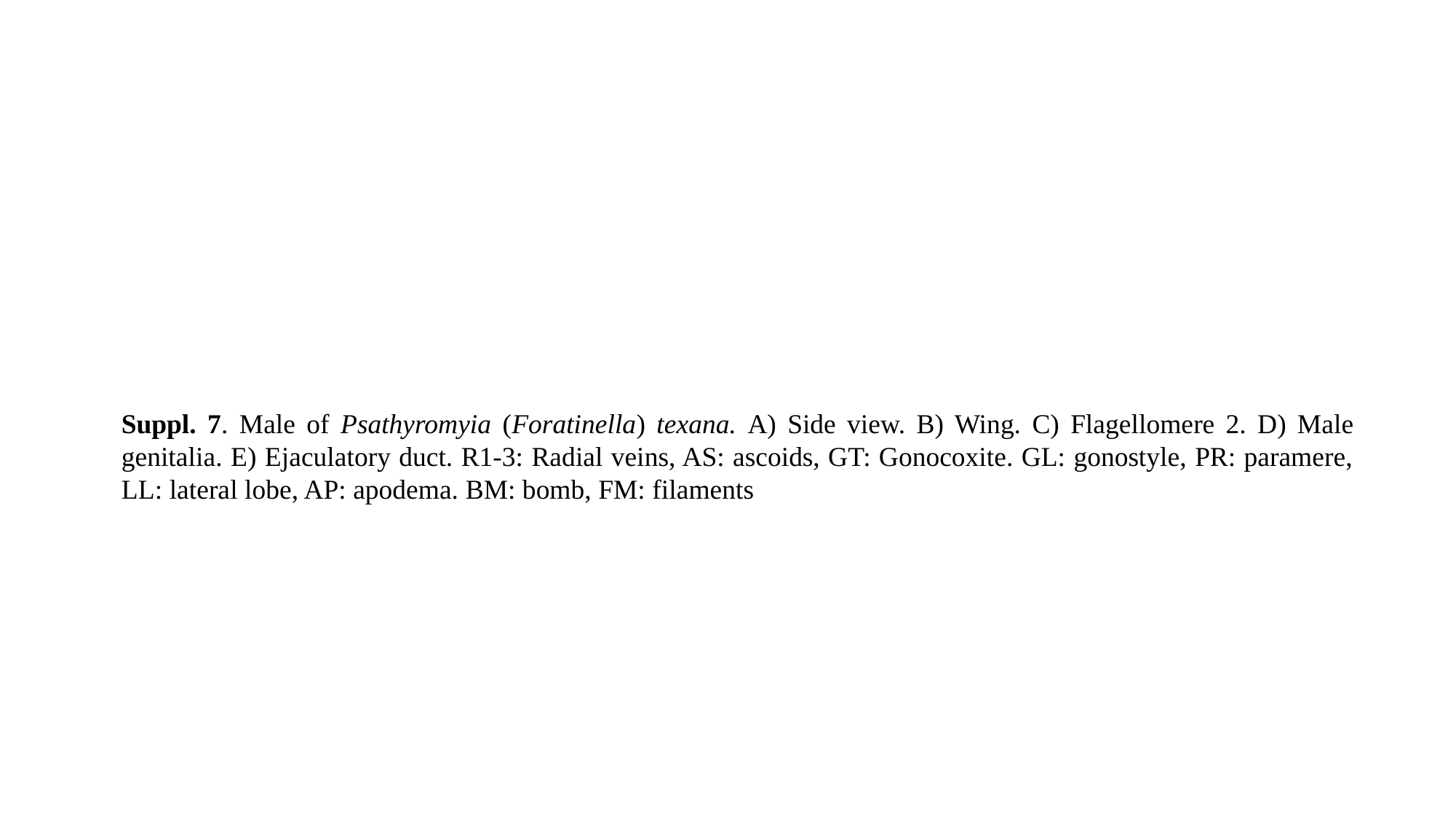

Suppl. 7. Male of Psathyromyia (Foratinella) texana. A) Side view. B) Wing. C) Flagellomere 2. D) Male genitalia. E) Ejaculatory duct. R1-3: Radial veins, AS: ascoids, GT: Gonocoxite. GL: gonostyle, PR: paramere, LL: lateral lobe, AP: apodema. BM: bomb, FM: filaments
