# Supplementary material for: Leishmaniasis Transmission Risk at the Forest‐Peridomestic Interface in an Area of Southern Sinaloa, Mexico: Entomological, Molecular, and Climatic Evidence
Source: J Parasitol Res. 2026 Jun 16;2026:5071505. doi: 10.1155/japr/5071505 (PMC13270774; doi:10.1155/japr/5071505)
Supplement: Supplementary file 8 — Supporting Information 8. Climatic variables recorded at each sampling. [file JAPR-2026-5071505-s003.docx]

**Suppl. 4**

Climatic variables recorded at each sampling period during the study (July 2023 – June 2025), Sinaloa, Mexico. Data correspond to regional monthly averages from the nearest meteorological station shared across all four sampling localities.

|  |  |  | **Climatic variables** | | | |  |
| --- | --- | --- | --- | --- | --- | --- | --- |
| **Sampling event** | **Date** | **Season** | **Mean temperature (°C)** | **Precipitation (mm)** | **Relative humidity (%)** | **Wind speed (km/h)** | **Climatic notes** |
| S1 | Jul 2023 | Rainy | 30.5 | 128.7 | 64.6 | 6.4 | Moderate rainfall; high temp. |
| S2 | Sep 2023 | Rainy | 30.0 | 224.9 | 67.2 | 3.2 | Peak rainfall; low wind speed |
| S3 | Nov 2023 | Dry | 27.1 | 6.8 | 78.6 | 0.1 | Transition to dry; high humidity |
| S4 | Jan 2024 | Dry | 23.6 | 0.0 | 76.0 | 0.0 | Minimum temp.; no precipitation |
| S5 | Mar 2024 | Dry | 24.5 | 0.0 | 78.7 | 1.7 | Dry; humidity remains elevated |
| S6 | May 2024 | Dry | 27.5 | 0.0 | 78.9 | 6.9 | Pre-rainy season; max. wind speed |
| S7 | Jul 2024 | Rainy | 30.9 | 67.2 | 64.8 | 6.6 | Maximum temp. recorded; rainy onset |
| S8 | Sep 2024 | Rainy | 28.9 | 354.2 | 66.9 | 2.7 | Highest precipitation of study period |
| S9 | Nov 2024 | Dry | 28.4 | 5.2 | 79.1 | 0.1 | Max. humidity; near-zero wind |
| S10 | Jan 2025 | Dry | 22.8 | 0.0 | 76.5 | 0.1 | Lowest temp. of study period |
| S11 | Mar 2025 | Dry | 27.5 | 0.0 | 77.2 | 2.3 | Dry; moderate humidity |
| S12 | Jun 2025 | Rainy | 29.4 | 1.0 | 71.5 | 7.0 | Rainy season onset; max. wind speed |
| **Descriptive statistics across all sampling events (n = 12)** | | | | | | | |
| Minimum | | | 22.8 | 0.0 | 64.6 | 0.0 |  |
| Maximum | | | 30.9 | 354.2 | 79.1 | 7.0 |  |
| Mean ± SD | | | 27.6 ± 2.7 | 65.7 ± 115.0 | 73.3 ± 5.9 | 3.1 ± 2.9 |  |
| Total precipitation (mm) | | | — | 788.0 | — | — |  |
| **Rainy season events (n = 5)** | | | **29.9 ± 0.8** | **155.2 ± 138.5** | **67.0 ± 2.8** | **5.2 ± 1.9** |  |
| **Dry season events (n = 7)** | | | **25.9 ± 2.2** | **1.7 ± 2.6** | **77.9 ± 1.3** | **1.6 ± 2.5** |  |

SD = standard deviation. Season classification follows the Pacific coast of Sinaloa, Mexico: rainy season (June–October); dry season (November–May).

Climatic data represent monthly regional averages shared across all four sampling localities (Tecualilla, Ej. La Campana, Palmillas, and Copales).

Precipitation values of 0.0 indicate months with no recorded rainfall at the reference station.
